# Biological phosphorylation of an Unnatural Base Pair (UBP) using a *Drosophila melanogaster* deoxynucleoside kinase (DmdNK) mutant

**DOI:** 10.1371/journal.pone.0174163

**Published:** 2017-03-21

**Authors:** Fei Chen, Yuan Zhang, Ashley B. Daugherty, Zunyi Yang, Ryan Shaw, Mengxing Dong, Stefan Lutz, Steven A. Benner

**Affiliations:** 1 CAS Key Laboratory of Genome Sciences & Information, Beijing Institute of Genomics, Chinese Academy of Sciences, Beijing, China; 2 University of Chinese Academy of Sciences, Beijing, China; 3 Foundation for Applied Molecular Evolution (FfAME), Alachua, Florida, United States of America; 4 College of Chemistry, Beijing Normal University, Beijing, China; 5 Department of Chemistry, Emory University School of Medicine, Atlanta, Georgia, United States of America; Istituto di Genetica Molecolare, ITALY

## Abstract

One research goal for unnatural base pair (UBP) is to replicate, transcribe and translate them *in vivo*. Accordingly, the corresponding unnatural nucleoside triphosphates must be available at sufficient concentrations within the cell. To achieve this goal, the unnatural nucleoside analogues must be phosphorylated to the corresponding nucleoside triphosphates by a cascade of three kinases. The first step is the monophosphorylation of unnatural deoxynucleoside catalyzed by deoxynucleoside kinases (dNK), which is generally considered the rate limiting step because of the high specificity of dNKs. Here, we applied a *Drosophila melanogaster* deoxyribonucleoside kinase (DmdNK) to the phosphorylation of an UBP (a pyrimidine analogue (6-amino-5-nitro-3-(1’-b-d-2’-deoxyribofuranosyl)-2(1H)-pyridone, **Z**) and its complementary purine analogue (2-amino-8-(1’-b-d-2’-deoxyribofuranosyl)-imidazo[1,2-a]-1,3,5-triazin-4(8H)-one, **P**). The results showed that DmdNK could efficiently phosphorylate only the dP nucleoside. To improve the catalytic efficiency, a DmdNK-Q81E mutant was created based on rational design and structural analyses. This mutant could efficiently phosphorylate both dZ and dP nucleoside. Structural modeling indicated that the increased efficiency of dZ phosphorylation by the DmdNK-Q81E mutant might be related to the three additional hydrogen bonds formed between E81 and the dZ base. Overall, this study provides a groundwork for the biological phosphorylation and synthesis of unnatural base pair *in vivo*.

## Introduction

Genetic alphabet expansion with unnatural base pairs (UBPs) represents an important embranchment of chemical synthetic biology and is a key focus of synthetic biology [1]. Over the past twenty-five years, scientists have made considerable progress in genetic alphabet expansion by synthesizing different types of nucleotide analogs. Thus far, different UBPs designed based on different hydrogen bonding patterns and/or shape complementarities have been successfully developed by Benner [2–5], Hirao [6–9] and Romesberg [9–14]. Among them, three UBPs (Z-P, developed by Benner SA; Ds-Px, developed by Hirao I; and 5SICS-NaM, developed by Romesberg FE) have been particularly successful, and the DNA fragments containing these UBPs can be efficiently and faithfully amplified *in vitro* by PCR (fidelity >99%) [3, 8, 10].

The final goal of research on the expanded genetic alphabet is to replicate, transcribe and translate UBPs *in vivo*, which would lay the foundation for a semi-synthetic organism with an increased potential for information storage and retrieval [1]. In May 2014, Romesberg FE and his colleagues reported an engineered semi-synthetic bacterium whose genetic material included a d5SICS-dNaM unnatural base pair [14]. This bacterium could continually replicate an UBP under the supply of artificial molecular building blocks (d5SICS and dNaM). This study was a breakthrough in chemical synthetic biology because it was the first to replicate the artificially expanded genetic alphabet from *in vitro* to *in vivo*.

**Fig 1:**
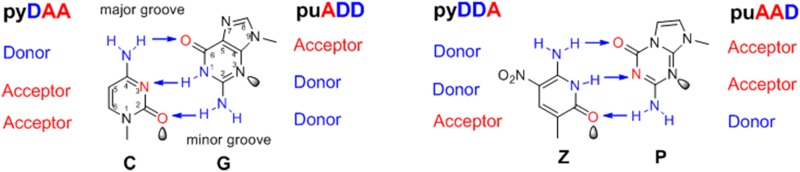
Structure of the natural C:G and unnatural Z:P base pairs. The natural C:G and unnatural Z:P base pairs all fit the Watson-Crick geometry, with large purines (or purine analogs, both indicated by “pu”) pairing with small pyrimidines (or pyrimidine analogs, both indicated by “py”) joined by hydrogen bonds. The hydrogen-bonding donor (D) and acceptor (A) groups are listed from the major to the minor groove. The arrow indicates the hydrogen bond between the donor and acceptor. Unshared pairs of electrons (or “electron density”) presented to the minor groove are shown by the shaded lobes.

Herein, the replication of the d5SICS-dNaM UPB occurred only once in the whole genome of this semi-synthetic organism. Even *in vitro*, only **Z** (6-amino-5-nitro-3-(1’-β-D-2’-deoxyribofuranosyl)-2(1H)-pyridone) and its complementary unnatural base **P** (2-amino-8-(1’-β-D-2’-deoxyribofuranosyl)-imidazo[1,2-a]-1,3,5-triazin-4(8H)-one)) (Fig 1) can be used in the PCR amplification of templates containing more than two consecutive unnatural bases with high fidelity (>99%) [3], although this behavior might have been related to the design of the Z-P UBP, which accounts for shape complementarity and hydrogen bond interactions [1]. In addition, the Z-P UBP also displayed many other advantages, such as good orthogonality and polymerase tolerance (almost all commercial polymerases will likely accept the Z-P UBP) [3]. Research on the Z-P UBP has been expanded to many areas, including nucleic acid testing and the development of a six-letter DNA aptamer [5, 15].

Over the past decade, significant research progress has been made on Z-P UBPs *in vitro* [1–5]; thus, a higher research goal should be set: introduce the Z-P UBP into natural living cells. To survive *in vivo*, the corresponding unnatural nucleoside triphosphates must be available at sufficient concentrations within the cell for DNA replication. Nucleoside triphosphates are known to be unstable and difficult to absorb by cells [16]. Thus, the unnatural nucleoside may be a better option. Thus, the unnatural nucleoside analogues must be recognized *in vivo* and phosphorylated to the corresponding nucleoside triphosphates by a cascade of three kinases: deoxynucleoside kinases (dNK), deoxynucleoside monophosphate kinases (dNMPK), and nucleoside diphosphate kinases (dNDPK) [17]. The first step is the monophosphorylation of deoxynucleoside catalyzed by dNKs. Because of the well-known high specificity of dNKs, this step is generally considered the rate limiting step [18]. The monophosphates are then further phosphorylated by the other two kinases with lower selectivity, which ultimately generates the unnatural deoxynucleoside triphosphates for DNA replication. If the endogenous kinases cannot phosphorylate the nucleoside analogues, we need to exogenously transfect the appropriate kinase gene into the cell, which is similar to action of certain nucleic acid drugs [19].

**Fig 2:**
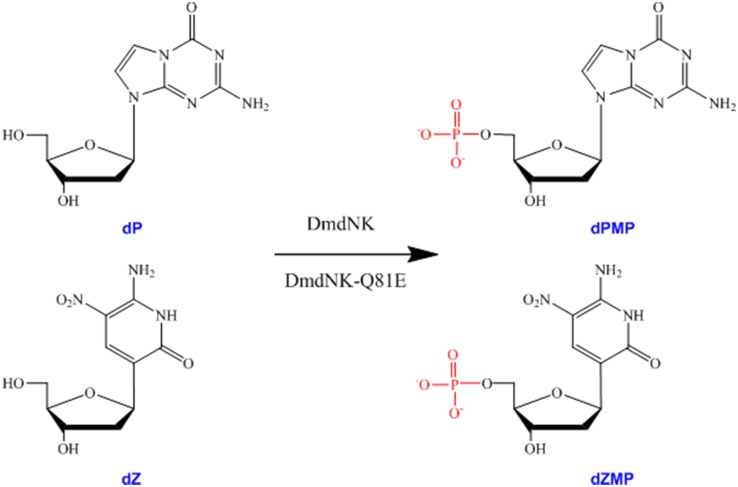
Biological phosphorylation of the dZ-P unnatural nucleosides by DmdNK and DmdNK-Q81E. dZ and dP are monophosphated and turned into the corresponding dZMP and dPMP by DmdNK and DmdNK-Q81E.

We have already attempted the phosphorylation of the Z-P UBP [20]. The *E*. *coli* NDPK has been shown to phosphorylate the Z-P nucleoside diphosphate. However, the endogenous *E*. *coli* dNKs could not efficiently phosphorylate the Z-P nucleosides (data not shown). Therefore, we employed *Drosophila melanogaster* deoxyribonucleoside kinase (DmdNK) [21–23] and tested its ability to phosphorylate the Z-P nucleoside (Fig 2). Previous studies have shown that DmdNK is a type of multisubstrate deoxyribonucleoside kinase that is capable of phosphorylating many nucleoside analogs [22–24]. Our results showed that DmdNK was only able to efficiently phosphorylate the dP. To obtain a high catalytic efficiency for dZ, a Q81E DmdNK mutant (DmdNK-Q81E) was designed based on structural analysis, and showed high catalytic efficiency for dZ. Further structural modeling indicated that the increased efficiency of DmdNK-Q81E in the phosphorylation of dZ may be related to the three additional hydrogen bonds formed between E81 and the dZ base. Therefore, this study provides a groundwork for the biological phosphorylation and synthesis of an unnatural base pair.

## Materials and methods

### Materials

The plasmid pDIM-DmdNK [25], the expression plasmid pET14b, and the host strains *E*. *coli* DH5α and BL21 (DE3) were obtained from our laboratory stocks. The enzymes and reagents for DNA manipulations were purchased from New England Biolabs. The polyethyleneimine-cellulose F TLC plates were purchased from Merck. The two unnatural deoxyribonucleosides, dZ and dP, were synthesized as previously described [4]. The two standard deoxyribonucleosides (deoxycytidine (dC) and deoxyguanosine (dG)) were purchased from Sigma.

### Mutagenesis, protein expression and purification

The DmdNK gene was amplified from the plasmid pDIM-DmdNK [25] via PCR (Primer 1: 5’-CTATCATATGGCGGAGGCAGCATCCTGTG-3’; Primer 2: 5’-CTCACTAGTTCATCTGGCGACCCTCTG-3’), and the NdeI and SpeI restriction sites were located at the 5’ and 3’ ends, respectively. Then, the PCR product was cloned into the expression plasmid pET14b. The constructed plasmid pET14b-DmdNK was confirmed by DNA sequencing. Using the parental plasmid pET14b-DmdNK, the expression plasmid of the DmdNK variant Q81E was constructed. The Q81E mutation was introduced by site-directed mutagenesis (C > G mutation, marked with a bold underlined letter) via overlap extension PCR (Primer 1: 5'-TAATACGACTCACTATAGGG-3'; Primer 2: 5’-TGCCCTTT**G**AGAGTTATGTC-3’; Primer 3: 5'-GCTAGTTATTGCTCAGCGG-3').

For expression, the constructed plasmids were transformed into the *E*. *coli* BL21 (DE3) expression strains. A single colony was selected and grown in 100 ml of LB media containing 100 mg/ml ampicillin with shaking (200 rpm) at 37°C overnight. The cultures were diluted to 1:50 in fresh antibiotic-containing LB media and shaken at 37°C until an *OD*_*600*_ of 0.6 was achieved. Protein expression was then induced by the addition of IPTG (0.1 mM) and incubation for ~5 h at 25°C.

For purification, the cells were harvested by centrifugation at 4,000 rpm for 30 min. Cell pellets were re-suspended in buffer A (50 mM potassium phosphate, 300 mM NaCl, 1 mM β-mercaptoethanol, 10% glycerol, pH 8.0) supplemented with benzonase nuclease (Novagen), lysozyme (Amresco), and protease inhibitor cocktail (Novagen) and then disrupted by ultrasonication in an ice bath. The lysate was centrifuged at 16,000 rpm for 30 min. Then, the supernatant was applied to a Chelating Sepharose Fast Flow column (GE Healthcare), which had been immobilized with Ni^2+^ and equilibrated with buffer A. After a wash step with 5 column volumes of buffer A containing 20 mM imidazole, the target protein was eluted with buffer A containing 150 mM imidazole. Fractions containing the target protein were identified by SDS-PAGE, dialyzed overnight against storage buffer (50 mM Tris-HCl, 150 mM NaCl, 1 mM β-mercaptoethanol, 10% glycerol, pH 8.0), and then stored at −20°C in aliquots. The protein concentration was determined by the Bradford Protein Assay. Chromatography experiments were performed on an AKTA Purifier-10 system.

### Phosphorylation kinetics assay of DmdNK and DmdNK-Q81E

The adenosine 5’-triphosphate transfer assay was conducted to measure the enzymatic activities under multiple turnover conditions [24, 26]. Analyses were performed with varying concentrations of nucleoside substrates in 50 mM Tris-HCl (pH 7.6), 100 mM KCl, 5 mM MgCl_2_, 0.5 mg/mL BSA, 1 mM dithiothreitol (DTT), 10 μCi [γ ^32^P]-ATP and 100 μM unlabeled ATP. The reaction was initiated by adding a fixed concentration of enzyme followed by incubating at 37°C and terminated by heating at 95°C for 5 min. Aliquots were collected at various times and applied to polyethyleneimine-cellulose F TLC plates (Merck). Chromatography was performed with isobutyric acid:NH_4_OH: H_2_O (66:1:33) (v/v) as the mobile phase for 8–12 h. The products were visualized by autoradiography. The apparent *K*_*M*_ and *V*_*max*_ values were determined according to the Eadie-Hofstee plot. All of the experiments were performed in triplicate using the same batch of purified enzymes. Data represent the mean ± SD (n = 3).

## Results

The phosphorylation activities of DmdNK and DmdNK-Q81E with different deoxyribonucleosides (dC / dG and dZ / dP) were examined under multiple turnover conditions. The kinetic parameters *k*_*cat*_ and *K*_*M*_ were derived from the Eadie-Hofstee plots (Table 1), and the catalytic efficiency was determined by *k*_*cat*_/*K*_*M*_ [24, 26].

**Table 1:** Kinetic parameters of DmdNK and its mutant DmdNK-Q81E with different substrates.

	Substrate	*K*_*M*_ (μM)	*k*_*cat*_ (min^-1^)	*k*_*cat*_*/K*_*M*_ (M^-1^ min^-1^)
**DmdNK**	dG	430.7±47.2	77.0 ±8.0	1.8 ×10^5^
	dC	8.7±0.7	86.0 ±4.0	9.9×10^6^
	dP	90.0 ±10.9	49.0 ±2.3	5.4 ×10^5^
	dZ	101.7±4.0	0.35±0.02	3.5 × 10^3^
**DmdNK-Q81E**	dG	1612.9±44	43.7±4	2.7 ×10^4^
	dC	11.3±0.4	126±10	1.1 × 10^7^
	dP	21.7±5.8	83.9±16.5	3.9 × 10^6^
	dZ	0.68±0.15	4.0 ±0.8	5.9 × 10^6^

As shown in Table 1, the wild-type DmdNK exhibited high catalytic efficiency for the phosphorylation of its natural substrates dC and dG, which is consistent with previous reports [21–23]. However, the catalytic efficiency of DmdNK varied for the two unnatural substrates. For the dG analog dP, DmdNK retained high catalytic efficiency (*k*_*cat*_/*K*_*M*_ = 5.4 ×10^5^ vs. 1.8 ×10^5^ M^-1^ min^-1^ for dP and dG, respectively), whereas for the dC analog dZ, its phosphorylation was approximately 3,000-fold less efficient than that for dC (*k*_*cat*_/*K*_*M*_ = 3.5 × 10^3^ vs. 9.9×10^6^ M^-1^ min^-1^ for dZ and dC, respectively). This difference was mainly because of a sharp decline in the *K*_*cat*_ value (*k*_*cat*_ = 0.35 vs. 86.0 min^-1^ for dZ and dC, respectively). Compared with dC, the affinity of DmdNK for dZ was also decreased more than 10-fold (*K*_*M*_ = 8.7 vs. 101.7 μM for dC and dZ, respectively). Previous research has shown that C-glycosides influence the enzymatic recognition and acceptance of dZ [27, 28].

To improve the catalytic efficiency of DmdNK towards dZ, the DmdNK mutant DmdNK-Q81E was designed and tested for its phosphorylation activity towards the unnatural nucleosides. As shown in Table 1, DmdNK-Q81E phosphorylated dZ and dP with a similarly high efficiency. Compared with the wild type, the phosphorylation efficiency of the mutant for dZ increased significantly (~1700-fold) (*k*_*cat*_/*K*_*M*_ = 5.9 × 10^6^ vs. 3.5 × 10^3^ M^-1^ min^-1^ for DmdNK-Q81E and DmdNK, respectively). The substitution of GLn81 with Glu resulted in a greater than 10-fold increase in catalytic rate (*k*_*cat*_ = 4.0 vs. 0.35 min^-1^ for DmdNK-Q81E and DmdNK, respectively) and an approximately 150-fold increase in affinity (*K*_*M*_ = 0.68 vs. 101.7 μM for DmdNK-Q81E and DmdNK, respectively). As for dP phosphorylation, the catalytic efficiency of the mutant was also increased compared with the wild type (*k*_*cat*_/*K*_*M*_ = 3.9 × 10^6^ vs. 5.4 × 10^5^ M^-1^ min^-1^ for DmdNK-Q81E and DmdNK, respectively) because of a slight increase in catalytic rate (*k*_*cat*_ = 83.9 vs. 49.0 min^-1^ for DmdNK-Q81E and DmdNK, respectively) and an approximately 4-fold decrease in affinity (*K*_*M*_ = 21.7 vs. 90.0 μM for DmdNK-Q81E and DmdNK, respectively).

## Discussion

The deoxyribonucleoside kinase from *Drosophila melanogaster* (DmdNK) is known to efficiently phosphorylate all four natural nucleosides and many nucleoside analogs [21–26]. Because of the broad substrate specificity and the high catalytic efficiency, the structure and function of DmdNK have been intensively investigated by many researchers. The formation of many complex crystal structures between DmdNK and its different natural/unnatural substrates or its feedback inhibitors have been reported [29–34]. Additionally, successful DmdNK mutants have been constructed to phosphorylate specific nucleoside analogs [24, 35–38].

Based on these advantages, we applied DmdNK to the biological phosphorylation of the Z-P UBP in this research. Our results showed that the wild-type DmdNK was able to efficiently phosphorylate the natural dC and dG and unnatural dP nucleosides, whereas the phosphorylation of the dZ nucleoside was less efficient. To overcome this obstacle, we engineered a DmdNK mutant by rational design. The crystal structure of the DmdNK:dC complex has been reported (PDB code: 1J90, 2VP5), which revealed that DmdNK has an α/β mixed architecture that consists of eight α-helices and a central five-strand parallel β-sheet [28, 32]. The dC nucleoside is located in a pocket near the C-terminus of the β-sheet. The cytosine base is packed among four polar residues (E52, Y70, Q81 and R105) and several hydrophobic residues (F111, W57, F80, M69, V84, M88, A110 and M118). The polar residue Q81 forms two hydrogen bonds to the base via N3 and N4. The other three polar residues are hydrogen bonded to the deoxyribose ring or water molecule. Herein, only Q81 was found to exhibit hydrogen bonds to the cytosine base; therefore, the decreased phosphorylation efficiency of the DmdNK for dZ may have resulted from the attenuated hydrogen bond interactions between the dZ base and the Gln81 residue of DmdNK. As shown in Fig 1, although the chemical structures of dC and dZ appear similar, N3 is a hydrogen acceptor in the cytosine base but a hydrogen donor in the Z base. Considering that the amide group of the Gln81 is also a hydrogen donor, the hydrogen bond (formed between N3 of the cytosine base and the amide group of the Gln81) no longer formed when the cytosine base was substituted with the Z base. Moreover, repulsion occurred between the N3 of the Z base and the amide group of the Gln81. According to the above speculation, we hypothesize that if Gln81 is replaced with the acidic residue, the corresponding repulsion may dissipate and additional hydrogen bonds between the Z base and the substituted residue may form. Glu is the best option because its chemical structure resembles that of Gln and it is an acidic residue.

**Fig 3:**
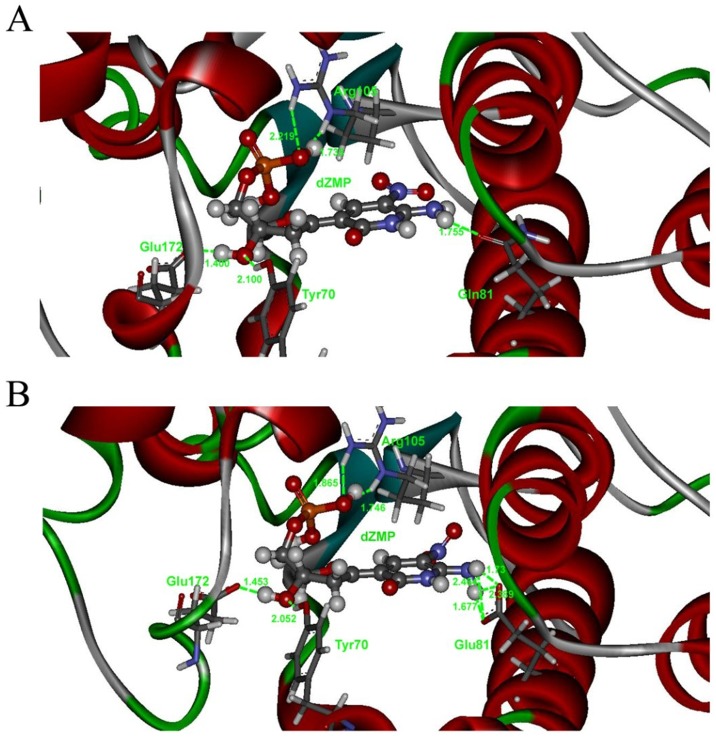
Detailed diagram of the hydrogen bonding interactions between dZ and DmdNK (a) or DmdNK-Q81E (b). The Gln81, C and G in the DmdNK:dC complex crystal structure (PDB code: 2VP5/2VP2) (28, 32) were manually modified to Glu, Z and P in the corresponding models. The hydrogen bonds are marked by green lines, and their distances are labeled in green numbers. The atoms are colored by element.

To further explore our hypothesis, we modeled the interactions between DmdNK/DmdNK-Q81E and the two unnatural deoxyribonucleosides based on the DmdNK:dC complex crystal structure (PDB code: 2VP5/2VP2). The Gln81, C and G in the experimentally determined crystal structures were manually modified to Glu, Z and P in the corresponding models. The modified structures were evaluated and optimized by Amber10 software. Then, the manually modified models describing the interactions between DmdNK/DmdNK-Q81E and the two unnatural deoxyribonucleosides were subject to energy minimization. The figures were generated by Discovery Studio Visualizer 3.5 (Accelrys Inc., San Diego, CA, USA, 2012). As shown in Fig 3, compared with only one hydrogen bond formed between the Z base and Gln81 in the DmdNK:dZ complex model, Glu81 has four hydrogen bonds that bind to the Z base in the DmdNK-Q81E:dZ complex model. In theory, the increased hydrogen bonds may lead to enhanced enzymatic affinity and a decline in *K*_*M*_ value (Table 1). Indeed, the substitution of Gln81 with Glu resulted in an approximately 150-fold increase in affinity and a greater than 10-fold increase in catalytic rate.

In this work, rational design and a detailed structure analysis were used to construct a mutant of DmdNK (DmdNK-Q81E) that could efficiently phosphorylate the unnatural deoxyribonucleosides dZ and dP. Therefore, this study provides a groundwork for the biological phosphorylation and synthesis of an unnatural base pair *in vivo*.

## Supporting information

S1 Fig Kinetic analysis of DmdNK with different substrates under multiple turnover conditions. (DOCX)

S2 Fig Kinetic analysis of Q81E with different substrates under multiple turnover conditions. (DOCX)
