# Supplementary material for: Biological phosphorylation of an Unnatural Base Pair (UBP) using a Drosophila melanogaster deoxynucleoside kinase (DmdNK) mutant
Source: PLoS One. 2017 Mar 21;12(3):e0174163. doi: 10.1371/journal.pone.0174163 (PMC5360312; doi:10.1371/journal.pone.0174163)
Supplement: S2 Fig — (DOCX) [file pone.0174163.s002.docx]

***Supporting information:***

**Biological Phosphorylation of an Unnatural Base Pair (UBP) using a *Drosophila melanogaster* Deoxynucleoside Kinase (DmdNK) Mutant**

Fei Chen^1, 2, 3¶ *^, Yuan Zhang^4¶^, Ashley B Daugherty^5^, Zunyi Yang^3^, Ryan Shaw^3^, Mengxing Dong^1,2^, Stefan Lutz^5^, Steven A Benner^3*^

**S2 Fig.**

**
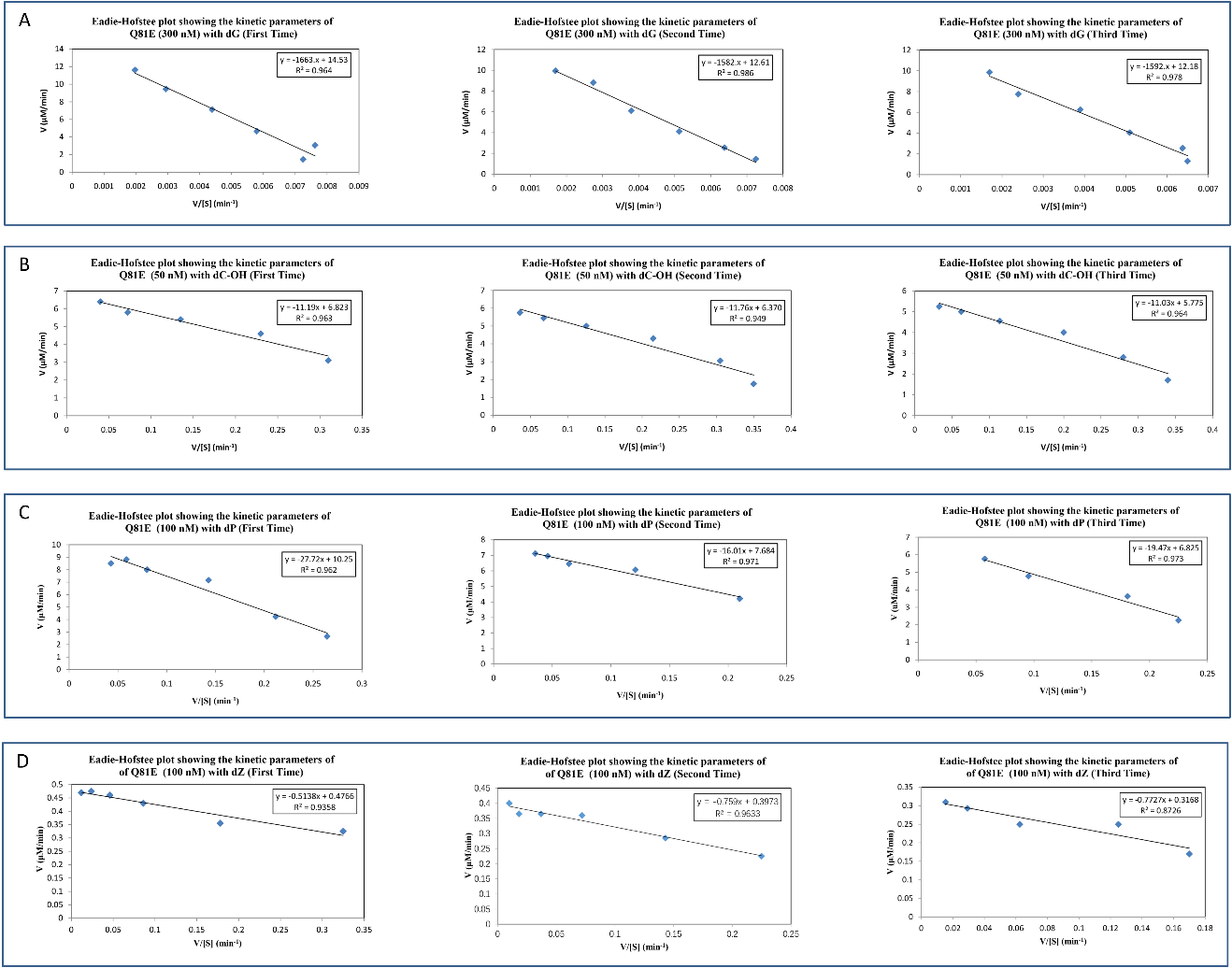
**

**S2 Fig. Kinetic analysis of Q81E with different substrates under multiple turnover conditions:** All reactions were performed with a fixed concentration of DmdNK and excess amount of the substrates (as indicated). The initial velocity (V) was determined by measuring the radioactivity of the bands corresponding to the substrates and products with a PhosphorImager (Quantity One 1-D Analysis Software, Bio-Rad). The initial velocities at each substrate concentration were used to generate an Eadie-Hofstee plot. The *V_max_* and *K_M_* values were deduced from the y intercepts and the negative slopes, respectively. The *k_cat_* was then given from the formula (*V_max_* = *k_cat_* [E]). All the data represent mean ± SD of three replicates. A: Analysis of multiple turnover kinetics of Q81E (300 nM) with dG (200, 400, 800, 1600, 3200 and 5850 μM) [Repeat triple times]. B: Analysis of multiple turnover kinetics of Q81E (50 nM) with dC (5, 10, 20, 40, 80 and 160 μM) [Repeat triple times]. C: Analysis of multiple turnover kinetics of Q81E (100 nM) with dP (1, 2, 5, 10, 20 and 40 μM) [Repeat triple times]. D: Analysis of multiple turnover kinetics of Q81E (100 nM) with dZ (1, 2, 4, 10, 20 and 40 μM) [Repeat triple times].
